# The importance of smoking cessation in pancreatitis

**DOI:** 10.1111/ans.17843

**Published:** 2022-11-17

**Authors:** Eunjong (Franklin) Han, Jonathan Koea, Chet Hammill, Sanket Srinivasa

**Affiliations:** ^1^ Upper GI Unit, Department of Surgery, North Shore Hospital University of Auckland Auckland New Zealand; ^2^ Section of HPB Surgery Washington University/ Barnes Jewish Hospital St Louis Missouri USA

Acute and chronic pancreatitis both cause significant morbidity.^1^ Recurrent episodes of acute pancreatitis, and a progression into its chronic form may lead to lifelong debilitating complications such as diabetes, chronic pain, and an increased risk of pancreatic cancer.^2^ Despite significant advancements in the treatment of acute, complicated pancreatitis,^1^ the primary prevention strategy for recurrent episodes has remained largely unchanged. The current prevention strategy is directed at identifying and eliminating the primary cause‐ usually alcohol or gallstones.^3^ Therefore, following episodes of acute, non‐gallstone pancreatitis, if diagnostic tests return negative results for the less common causes (e.g., hypercalcemia or hypertriglyceridemia), alcohol is deemed to be the likely culprit and patients are advised to abstain indefinitely.

Recent studies have shown that in addition to alcohol, tobacco smoking is just as, if not more of an impactful risk factor for non‐gallstone pancreatitis pathogenesis. Historically, studies attempting to establish a causal relationship between smoking and pancreatitis have yielded inconclusive results. Heavy alcohol use and tobacco smoking are often present together, and thus very difficult to study in isolation.^4^ Hence, cigarette smoking has been largely overlooked as a risk factor.

In contrast to historically held beliefs, a large, multicenter study conducted between 2000 and 2006 demonstrated smoking to be an independent risk factor for pancreatitis.^5^ As illustrated by Table 1, smoking status, when adjusted for age and sex, correlated with an increased risk of recurrent acute pancreatitis as well as chronic pancreatitis in a dose‐dependent manner. In contrast, the risk of alcohol consumption was demonstrated to be specific to just chronic pancreatitis, and only at very heavy consumption levels as defined by more than five drinks per day.^5^ Additionally, smoking was shown to increase the risk of chronic pancreatitis by two‐to‐three‐fold even amongst non‐alcohol drinkers, or light drinkers, thus accentuating its role as a risk factor independent of alcohol consumption.^5^ These study findings were confirmed by a recent meta‐analysis of prospective studies that showed that smoking is associated with a 49% and 93% increased risk of acute and chronic pancreatitis respectively.^6^ Similarly, a separate study identified tobacco smoking as an independent risk factor for all forms of non‐gallstone pancreatitis, namely acute, recurrent, and chronic pancreatitis, while alcohol was unexpectedly identified to have some protective effect at lower consumption levels.^7^ While this is highly controversial, the aggregation of recent data seems to suggest tobacco smoking to be an important risk factor, perhaps even more so than alcohol, in pancreatitis pathogenesis.

**Table 1:** Odds ratios (OR) as reported by the ‘The North American Pancreatitis Study 2’, showing associations of alcohol consumption and smoking with recurrent acute pancreatitis (RAP) and chronic pancreatitis (CP). OR with a *p*‐value <0.05 is in bold (5)

Alcohol use^†^	Recurrent acute pancreatitis		Chronic pancreatitis		Smoking status (pack‐years)
	Alcohol	Smoking	Alcohol	Smoking	
Abstainer or light	1	1	1	1	Never
Moderate	0.82	0.96	0.81	1.34	<12
Heavy	0.70	1.44	0.83	**2.15**	12–35
Very heavy	1.16	**1.91**	**3.10**	**4.59**	>35

^†^ Alcohol use is categorized based on a self‐reported questionnaire. The category definitions are reported in ‘The North American Pancreatitis Study 2’ (5).

Despite the epidemiological relationship evident between pancreatitis and smoking, the molecular mechanism linking the two is yet to be fully described. Studies involving animal models have identified possible mechanistic associations involving several tobacco components, namely nicotine and the tobacco‐specific nitrosamine 4‐(methylnitrosamino)‐1‐(3‐pyridyl)‐1‐ butanone (NNK).^8^ These chemicals appear to induce pathogenesis through its interaction with pancreatic enzymes, such as trypsinogen and chymotrypsinogen, housed inside zymogen granules of acinar cells.^8^ NNK has shown to cause a three‐fold increase in trypsinogen activation, and a five‐fold increase in chymotrypsinogen, with no reciprocal up‐regulation of its natural inhibitor known as pancreatic secretory trypsin inhibitor (PSTI).^9^ On the other hand, nicotine exposure leads to an increase in total cellular content of another pancreatic enzyme called amylase through its cellular retention and reduced secretion.^10^ Both components of tobacco smoke have been shown to induce histological changes consistent with pancreatitis, characterized by vacuolization, pyknotic nuclei and cytoplasmic swelling.^9^, ^10^ It has been postulated that the processes involving excessive enzyme activation and retention create a pancreatic environment conducive to an inflammatory insult that may lead to the development of pancreatitis.^9^, ^10^ However, as not all smokers develop pancreatitis, it is possible that the above mechanism interacts with other pre‐existing environmental or genetic co‐factors to lead to pancreatitis pathogenesis.^8^, ^11^

Despite the evident epidemiological and molecular associations between smoking and non‐gallstone pancreatitis, the effectiveness of smoking cessation in the prevention of future pancreatitis episodes is less established. However, given the vast health benefits to the individual, society, and the healthcare system; in addition to potential benefits to chronic and recurrent pancreatitis, smoking cessation interventions should always be offered. Alcohol abstinence advice is offered routinely at the end of each patients' acute illness, so this would be an ideal opportunity to also offer smoking cessation advice. This should be done with care and compassion, without insinuating them as moral failings, as doing‐so may elicit negative emotions leading to barriers that may hinder behavioural adjustment. We would thus encourage readers to approach this conversation with empathy, while also acknowledging the importance of smoking cessation as a key management principle of pancreatitis.

## Author contributions

**Eunjong (Franklin) Han:** Writing – original draft; writing – review and editing. **Jonathan Koea:** Supervision; writing – review and editing. **Chet Hammill:** Supervision; writing – review and editing. **Sanket Srinivasa:** Supervision; writing – original draft; writing – review and editing.
